# Thyroid Hormone Resistance Indices and Their Correlations With Insulin Resistance in Chinese Euthyroid Subjects

**DOI:** 10.1155/ije/5397686

**Published:** 2025-09-24

**Authors:** Ruixiang Cui, GuiHua Li, Ying Wei, Jia Liu, Ying Wang, Song Leng, Guang Wang

**Affiliations:** ^1^Department of Endocrinology, Beijing Chao-Yang Hospital, Capital Medical University, No. 8, Gongti South Road, Chaoyang District, Beijing 100020, China; ^2^Health Management Center, The Second Affiliated Hospital of Dalian Medical University, No. 467, Zhongshan Road, Shahekou District, Dalian 116023, Liaoning, China; ^3^Health Management Center, Beijing Chao-Yang Hospital, Capital Medical University, No. 8, Gongti South Road, Chaoyang District, Beijing 100020, China

**Keywords:** body mass index, euthyroid, insulin resistance, metabolic syndrome, sensitivity to thyroid hormones

## Abstract

**Aim:** This study aimed to analyze the relationship between sensitivity to thyroid hormones and insulin resistance (IR) in people with different levels of body mass index (BMI).

**Methods:** We included 32,478 euthyroid participants, and they were divided into three groups according to body mass index (BMI): normal weight (*n* = 20,200), overweight (*n* = 10,178), and obesity (*n* = 2100). We used the Thyroid Feedback Quantile-based Index (TFQI), the TSH index (TSHI), and the Thyrotroph T4 Resistance Index (TT4RI) to represent thyroid hormones sensitivity and used Triglyceride-Glucose (TyG) Index, the Atherogenic Index of Plasma (AIP), and The Metabolic Score for Insulin Resistance (METS-IR) to represent IR.

**Results:** In the BMI < 25 kg/m^2^ group, linear regression showed that TFQI, TSHI, and TT4RI were all positively correlated with TyG (*β* = 0.096, 0.089, and 0.089, respectively, all *p* < 0.001). Logistic regression showed that the odds ratios (ORs) for having high TyG in the highest quartiles of the TFQI, TSHI, and TT4RI were 1.430, 1.537, and 1.518, respectively (all *p* < 0.001), compared with the lowest quartiles of the TFQI, TSHI, and TT4RI. In the 25 ≤ BMI < 29.9 kg/m^2^ group, TFQI, TSHI, and TT4RI were positively associated with TyG (*β* = 0.071, 0.067, and 0.066, respectively, all *p* < 0.001) according to linear regression. The ORs for having high TyG in the highest quartiles of the TFQI, TSHI, and TT4RI were 1.269, 1.363, and 1.353, respectively (all *p* < 0.001) by logistic regression. In the BMI ≥ 30 kg/m^2^ group, positive correlations were found between the three thyroid indices and TyG (*β*_TFQI_ = 0.097, *β*_TSHI_ = 0.084, *β*_TT4RI_ = 0.083, all *p* < 0.01) by linear regression. The ORs for having high TyG in the highest quartiles of the TFQI, TSHI, and TT4RI were 1.298 (*p*=0.101), 1.454 (*p*=0.05), and 1.455 (*p*=0.05), respectively.

**Conclusions:** Reduced sensitivity to thyroid hormones was associated with high levels of IR in euthyroid adults of normal weight and overweight. In obese patients, no significant correlation was found between sensitivity to thyroid hormones and IR.

## 1. Introduction

Insulin resistance (IR) is the state of decreased sensitivity and reactivity to the effects of insulin. Numerous metabolic disorders, including diabetes, hyperlipidemia, hyperuricemia, and polycystic ovarian syndrome, have been found to be caused by IR [1–4]. Additionally, it was shown to be linked to neuropsychiatric disorders and cardiovascular adverse events [1, 5]. In clinical practice, therefore, early identification of IR is quite meaningful to prevent further progression of correlative diseases.

Thyroid hormones (THs) operate as a vital regulator of metabolism. The relationship between THs and IR has been extensively studied, though fully consistent conclusions have not been drawn. For instance, the results of two studies that examined the relationship between free thyroxine (FT4) and the homeostasis model assessment of IR (HOMA-IR) index differed, finding no significant correlation and a negative correlation, respectively [6, 7]. In order to better reflect the status and function of the whole thyroid system, some new indexes including the Thyroid Feedback Quantile-based Index (TFQI), the TSH index (TSHI) [8], and the Thyrotroph T4 Resistance Index (TT4RI) [9] have been raised. In euthyroid humans, modest resistance to THs has been identified as a possible risk factor for metabolic diseases. For example, decreased TH sensitivity was found to be associated with metabolic dysfunction-associated fatty liver disease, type 2 diabetes (T2DM), and increased prevalence of metabolic syndrome [10, 11]. However, little has been done to study the relationship between sensitivity to THs and IR, which is the common pathogenesis of the above metabolic diseases. One previous study showed that in euthyroid adults with obesity, a decrease in central TH sensitivity was linked to higher IR. However, such relationship has not been verified in the general population with diverse body mass index (BMI) [12].

The gold standard of measuring IR is the hyperinsulinemic-euglycemic camp (HIEC), which is not feasible and economical enough to calculate IR in clinical practice [13]. There are several non-insulin-based, newly raised, and well-established indexes to represent IR in our study, including the Triglyceride-Glucose (TyG) index [14], the Atherogenic Index of Plasma (AIP) index [15], and the Metabolic Score for Insulin Resistance (METS-IR) index [16]. TyG showed more reliability than HOMA-IR in forecasting metabolic syndrome and has also been identified as a reliable indicator of diabetes and cardiovascular diseases [17]. AIP was initially put forward as a predictor for atherosclerotic plaque progression and adverse cardiovascular events [15]. Over the years, researchers gradually noticed its association with IR and IR-related metabolic diseases [18]. METS-IR index has also been proven to have a good correlation with HOMA-IR and predictive value for diabetes [16].

This study aimed to explore the BMI-specific relationship between sensitivity to THs and IR in the euthyroid participants. We assessed the sensitivity to THs in euthyroid individuals using the TFQI, TSHI, and TT4RI indices. We evaluated the level of IR by the TyG, AIP, and METS-IR indexes.

## 2. Materials and Methods

### 2.1. Study Population

Participants in this study must be at least 18 years old and must have had a routine physical examination performed at Beijing Chao-Yang Hospital's health medical center between April 2016 and December 2022. We did not include participants with known abnormal thyroid function, history of thyroid disorders or antithyroid therapy, hormone replacement therapy (corticosteroids or THs), use of thyroid function-influencing medications (such as amiodarone), severe renal or hepatic dysfunction, systemic inflammation, pregnancy, and cancer. Ultimately, 32,478 participants were included in our study. The Beijing Chao-Yang Hospital Ethics Committee approved the study procedure, and all subjects submitted informed consent in written form.

### 2.2. Data Collection

After an overnight fast, blood samples were collected from the vein. Serum thyroid-stimulating hormone (TSH), FT4, and free triiodothyronine (FT3) were measured by electrochemiluminescence immune assay using an Abbott Architect i2000 (Abbott Diagnostics, Abbott Park, IL, USA) as previously described [19]. Total cholesterol (TC), triglyceride (TG), high-density lipoprotein cholesterol (HDL-C), low-density lipoprotein cholesterol (LDL-C), fasting blood glucose (FBG), uric acid (UA), and hemoglobin A1c (HbA1c) were measured as previously described [20].

### 2.3. Definitions and Calculations of Variables

The World Health Organization (WHO) standard stated that a BMI of > 25 kg/m^2^ was considered overweight, and a BMI of ≥ 30 kg/m^2^ was considered obese [21]. Euthyroid was defined as TSH0.55 (0.55–4.78 μIU/mL), FT4 (0.89–1.76 ng/dL), and FT3 (2.3–4.2 pg/mL) within the reference ranges. Systolic blood pressure (SBP) of 140 mmHg or diastolic blood pressure (DBP) of 90 mmHg was the threshold for hypertension [22]. We denoted an estimated glomerular filtration rate (eGFR) of less than 30 mL/min/1.73 m^2^ as severe renal impairment. Transaminase levels ≥ 120 U/L were considered indicative of severe hepatic impairment. Since there was no common definition of a high value range of the three IR indexes, we defined the upper quartiles of the three indexes as high TyG, AIP, and METS-IR levels, respectively. TFQI, TSHI, and TT4RI were divided into four quartiles groups stratified by BMI.

Central indices of TH sensitivity were calculated with the following formulas: TFQI = cumulative distribution function (cdf) FT4 − (1−cdf TSH) [10]. Positive numbers suggested a lesser central sensitivity to the change in FT4, whereas negative values suggested a higher central sensitivity. TSHI = LnTSH (μIU/mL) + 0.1345 × FT4 (pmol/L) [8]. TT4RI = FT4 (pmol/L) × TSH (μIU/mL) [9]. Higher readings for TSHI and TT4RI suggested a decreased central sensitivity to THs. The formulas for indexes representing IR are as follows: TyG = ln[fasting plasma glucose (mg/dL) ∗ TG (mg/mL)/2] [14]; AIP = log (TG/HDL-C) [15]; METS-IR = Ln[(2 × fasting glucose (mg/dL)) + fasting TG (mg/dL)] × BMI (kg/m^2^))/(Ln[HDL-C (mg/dL)]) [16]. Greater values of these three indexes indicated higher levels of IR.

### 2.4. Statistical Analysis

Variables were presented as means ± standard deviations. Categorical variables were expressed as the quantity of events and percentages. Analysis of variance (ANOVA) was used to examine the significance of difference between mean values of TyG and AIP indexes across the quartiles of TH indices. Kruskal–Wallis test was employed to assess the significance of the variation in METS-IR index across the quartiles of TH indices. Age, sex, BMI, HbA1c, UA, and hypertension were adjusted, and then multivariate linear regression analysis and logistic regression analysis were performed to figure out the relationships between IR and thyroid system markers. The standardized coefficient *β* and *t* value were used to express the linear regression analysis results. The logistic regression analysis yielded odds ratios (OR) and 95% confidence intervals (CI). R 4.1.2 was used to perform all data analysis. A two-tailed *p* value < 0.05 was deemed statistically significant.

## 3. Results

### 3.1. Characteristics of the Study Population

Table 1 displays the clinical features of the research participants. This study recruited 32,478 euthyroid adults with a mean age of 43.2 years old. Participants were divided into three groups based on their BMI: the BMI < 25 kg/m^2^ group, the 25 kg/m^2^ ≤ BMI < 29.9 kg/m^2^ group, and the BMI ≥ 30 kg/m^2^ group. Each group contained 20,200, 10,178 and 2100 people, respectively. It can be observed that 85% of the entire study population suffered from hypertension. The overall mean levels of TyG, AIP, and METS-IR were 8.42, −0.12, and 35.0, respectively. TFQI, TSHI, and TT4RI had overall average levels of 0.011, 1.91, and 19.4, respectively.

### 3.2. Association of Sensitivity to TH Indices With TyG, AIP, and METS-IR Levels by Linear Regression Analysis

Table 2 shows TyG, AIP, and METS-IR levels by quartiles of indices of TH sensitivity. Generally, participants with higher thyroid sensitivity indices tend to have higher TyG, AIP, and METS-IR levels. The levels of the three indexes increased in a linear fashion over the TFQI, TSHI, and TT4RI quartiles. In the BMI < 25 kg/m^2^ group, both TyG and AIP increased in a linear fashion over the TFQI, TSHI, and TT4RI quartiles (all *p* < 0.001). In the 25 ≤ BMI < 29.9 kg/m^2^ group, TyG rose linearly over the TFQI, TSHI, and TT4RI quartiles (all *p* < 0.01), while AIP grew linearly only over the TFQI quartiles (*p* < 0.001). In the BMI ≥ 30 kg/m^2^ group, TyG, AIP, and METS-IR all rose above the TFQI quartiles in a linear pattern (all *p* < 0.01).

We next conduct linear regression analysis to further explore whether sensitivity to THs is independently associated with IR levels (Table 3). Age, sex, BMI, HbA1c, UA, and hypertension were adjusted. In the BMI < 25 kg/m^2^ group, all the TH indices were positively correlated with TyG and AIP (all *p* < 0.001). The same patterns were observed in METS-IR, except for the relationship between TFQI and METS-IR (*p*=0.175). In the 25 ≤ BMI < 29.9 kg/m^2^group, TSHI and TT4RI were positively associated with TyG (*β* = 0.067 and 0.066, respectively), AIP (*β* = 0.068 and 0.070, respectively), and METS-IR (*β* = 0.301 and 0.319, respectively) (all *p* < 0.001). TFQI presented a positive link with TyG (*β* = 0.071) and AIP (*β* = 0.053) (both *p* < 0.001). In the BMI ≥ 30 kg/m^2^ group, TFQI, TSHI, and TT4RI were positively correlated with TyG (*β* = 0.097, 0.084, and 0.083, respectively) (all *p* < 0.01), AIP (*β* = 0.081, 0.073, and 0.074, respectively) (all *p* < 0.05). No significant association was found between TFQI, TSHI, TT4RI, and METS-IR (*β* = 0.249, 0.231, and 0.238, respectively) (*p*=0.295, 0.254, and 0.248, respectively).

### 3.3. Relationship via Logistic Regression Analyses of TH Sensitivity Indices With High TyG, AIP, and METS-IR

**Figure 1:**
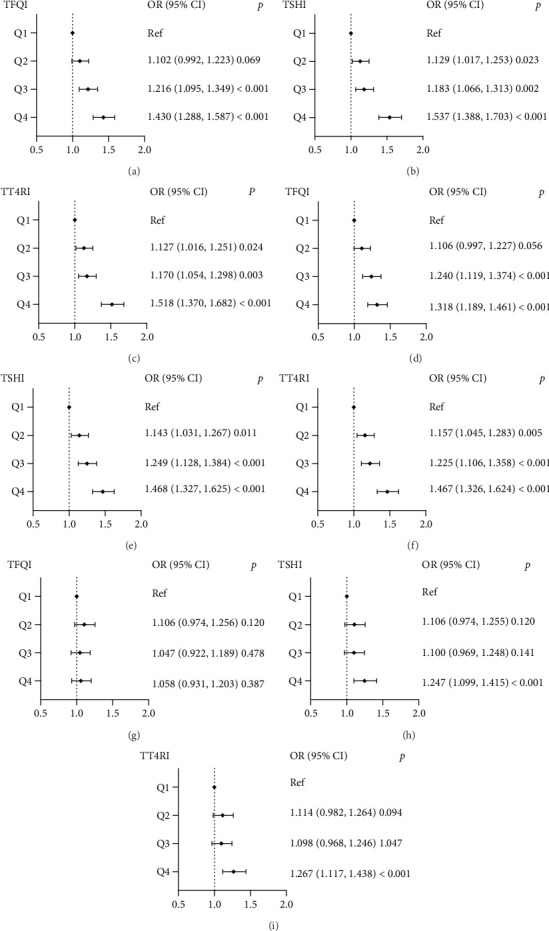
The forest maps of logistic regression analysis for the correlation between high IR levels (dependent variable) and quartiles of thyroid hormone sensitivity indices (independent variables) in the BMI < 25 kg/m^2^ group and the first quartile serves as the reference. (a–c) The ORs for high TyG levels across TFQI, TSHI, and TT4RI quartiles, respectively; (d–f) the ORs for high AIP levels across TFQI, TSHI, and TT4RI quartiles, respectively; (g–i) the ORs for high METS-IR levels across TFQI, TSHI, and TT4RI quartiles, respectively. BMI, body mass index; TyG, Triglyceride-Glucose Index; AIP, Atherogenic Index of Plasma; METS-IR, Metabolic Score for Insulin Resistance; TFQI, Thyroid Feedback Quantile-based Index; TSHI, TSH index; TT4RI, Thyrotroph T4 Resistance Index; age, sex, BMI, HbA1c, UA, and hypertension were adjusted.

The associations of TFQI, TSHI, and TT4RI quartiles with high TyG, AIP, and METS-IR levels in the BMI < 25 kg/m^2^ group are shown in Figure 1. The Q3 and Q4 TFQI levels demonstrated a growing positive correlation with elevated TyG levels in comparison to the Q1 TFQI level (Q3: OR 1.216, 95% CI, 1.095–1.349, Q4: OR 1.430, 95% CI, 1.288–1.587). The ORs of the Q2, Q3, and Q4 TSHI quartiles for high TyG levels were 1.129 (95% CI, 1.017–1.253), 1.183 (95% CI, 1.06–1.313), and 1.537 (95% CI, 1.388–1.703), respectively. Similarly, there was an increasing trend in the ORs of the Q3 and Q4 quantiles of TFQI, TSHI, and TT4RI for high AIP levels. As for METS-IR, only the OR of the highest TSHI and TT4RI quartiles for increased METS-IR level was statistically significant (OR 1.247, 95% CI, 1.099–1.415; OR 1.267, 95% CI, 1.117–1.438, respectively).

**Figure 2:**
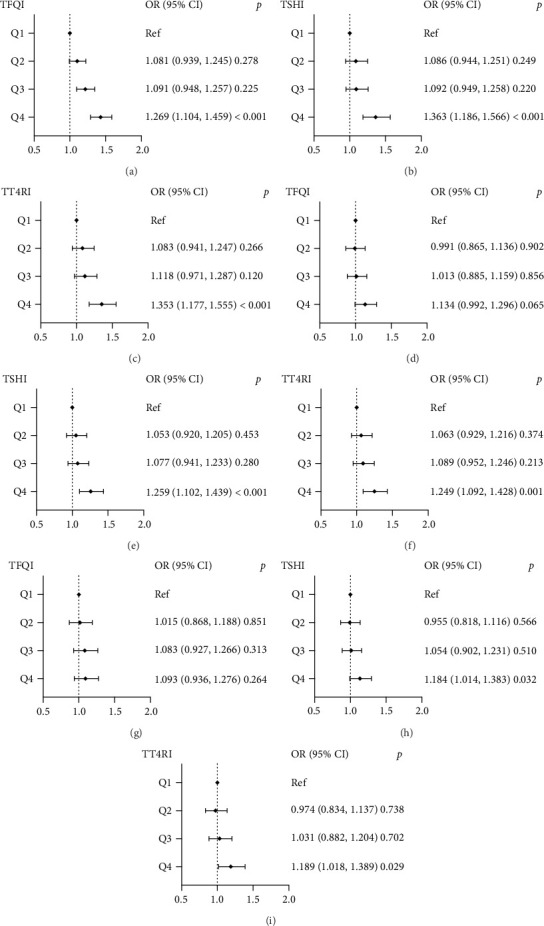
The forest maps of logistic regression analysis for the correlation between high IR levels (dependent variable) and quartiles of thyroid hormone sensitivity indices (independent variables) in the 25 ≤ BMI < 29.9 kg/m^2^ group and the first quartile serves as the reference. (a–c) The ORs for high TyG levels across TFQI, TSHI, and TT4RI quartiles, respectively; (d–f) the ORs for high AIP levels across TFQI, TSHI, and TT4RI quartiles, respectively; (g–i) the ORs for high METS-IR levels across TFQI, TSHI, and TT4RI quartiles, respectively. BMI, body mass index; TyG, Triglyceride-Glucose Index; AIP, atherogenic index of plasma; METS-IR, Metabolic Score for Insulin Resistance; TFQI, Thyroid Feedback Quantile-based Index; TSHI, TSH index; TT4RI, Thyrotroph T4 Resistance Index; age, sex, BMI, HbA1c, UA, and hypertension were adjusted.

The associations of TFQI, TSHI, and TT4RI quartiles with high TyG, AIP, and METS-IR levels in 25 kg/m^2^ ≤ BMI < 29.9 kg/m^2^ group are shown in Figure 2. Among the three indexes, only the ORs of the highest quartiles for elevated TyG, AIP, and METS-IR values were statistically significant compared to the lowest quartiles. The ORs (95% CI) of Q4 TFQI, TSHI, and TT4RI levels for elevated TyG levels were 1.269 (95% CI, 1.104–1.459), 1.363 (95% CI, 1.186–1.566), and 1.353 (95% CI, 1.177–1.555). For high AIP levels, the ORs (95% CI) of Q4 TSHI and TT4RI levels were 1.259 (95% CI, 1.102–1.439) and 1.249 (95% CI, 1.092–1.428), respectively. The ORs (95% CI) of Q4 TSHI and TT4RI levels for elevated METS-IR levels were 1.184 (95% CI, 1.014–1.38) and 1.189 (95% Cl, 1.018–1.389), respectively.

**Figure 3:**
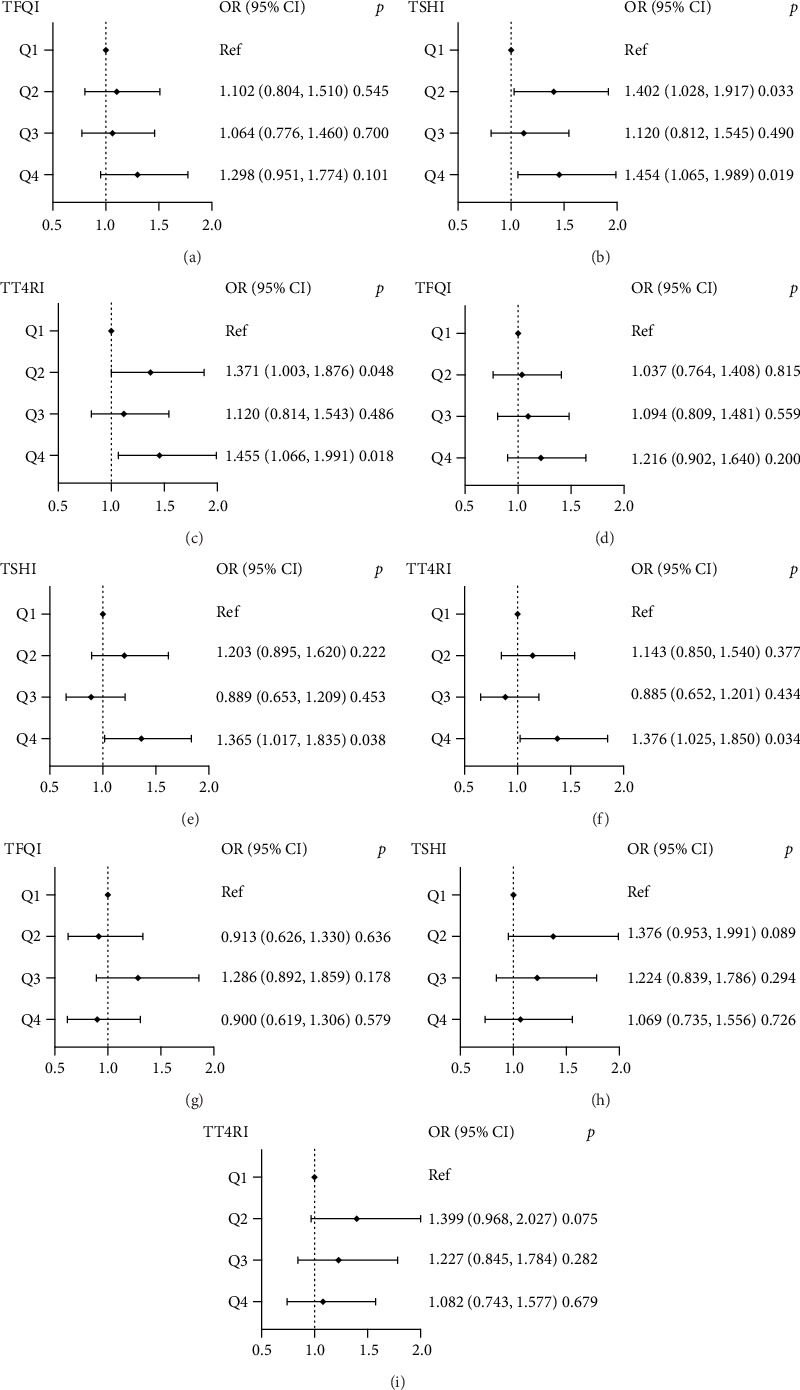
The forest maps of logistic regression analysis for the correlation between high IR levels (dependent variable) and quartiles of thyroid hormone sensitivity indices (independent variables) in the BMI ≥ 30 kg/m^2^ group and the first quartile serves as the reference. (a–c) The ORs for high TyG levels across TFQI, TSHI, and TT4RI quartiles, respectively; (d–f) the ORs for high AIP levels across TFQI, TSHI, and TT4RI quartiles, respectively; (g–i) the ORs for high METS-IR levels across TFQI, TSHI, and TT4RI quartiles, respectively. BMI, body mass index; TyG, triglyceride-glucose index; AIP, Atherogenic Index of Plasma; METS-IR, Metabolic Score for Insulin Resistance; TFQI, Thyroid Feedback Quantile-based Index; TSHI, TSH index; TT4RI, Thyrotroph T4 Resistance Index; age, sex, BMI, HbA1c, UA, and hypertension were adjusted.

Figure 3 shows the correlation between thyroid sensitivity indices and high IR indexes in the BMI ≥ 30 kg/m^2^ group. For high TyG levels, the ORs (95% CI) of Q2 and Q4 TSHI levels were 1.402 (95% CI, 1.028–1.917), 1.454 (95% CI, 1.065–1.989), and TT4RI:1.371 (95% CI, 1.003–1.876); 1.455 (95% CI, 1.066–1.991). The ORs (95% CI) of Q4 TSHI and TT4RI levels for raised AIP levels were 1.365 (95% CI, 1.017–1.835) and 1.376 (95% CI, 1.025–1.850), respectively.

## 4. Discussion

Our study investigated the association between thyroid sensitivity indexes and IR in euthyroid Chinese adults with different levels of obesity for the first time. We discovered that impaired sensitivity to THs, in other words, increased TFQI, TSHI, and TT4RI, was linked with higher levels of IR in the subjects of the normal weight or overweight group after adjusting for age, sex, BMI, HbA1c, UA, and hypertension. In the obese group, however, no significant association was found between sensitivity to THs and IR.

A prior study from Wei focused on obese populations and found that decrease in central THs sensitivity was linked to a higher level of adipose tissue IR [12]. There are also patients with normal weight who are metabolically unhealthy and suffer from IR-related glucolipid metabolism disorders [23]. Knowing the metabolic characteristics of these ostensibly healthy populations is also crucial. Interestingly, a positive link between FT4 and risk for metabolic syndrome was only found in nonobese subjects rather than obese ones [24]. It inspired us to explore the relationship between thyroid function and IR in various BMI levels, respectively. The effect of BMI on thyroid function has been investigated in several studies. A study showed that those with a higher BMI due to genetic predisposition also had higher FT3 levels [25]. Similarly, obesity was thought to be the cause of high TSH and FT4 in adolescents rather than a result of it [26]. Additionally, obesity is considered as the primary cause of IR [27]. BMI was also found to be positively associated with higher level of HOMA-IR [28]. Therefore, in this study, we stratify the subjects into three groups according to BMI. It is worth noting that waist circumference is a good indicator of central obesity. We adjusted for BMI rather than waist circumference in the regression analysis as BMI is a primary indicator for obesity screening and correlates with abdominal fat content as well [29].

Interestingly, we did not find a significant relationship between TH sensitivity indices and IR in the obese group, which seems to be contrary to the results of the prior study of Wei [12]. This may be due to the fact that different metrics were used to evaluate IR in the two studies. Wei et al. found the correlation only between thyroid sensitivity indexes and adipo-IR rather than and HOMA-IR or Matsuda index. The three indices selected in our study do not represent adipose tissue IR specifically but reflect systemic IR, which can also be influenced by IR in the liver or muscle. Notably, the level of obesity of participants included in Wei's study is quite serious, with an overall BMI of 38.4 kg/m^2^. The effect of thyroid function on IR in severely obese subjects may be different from that in patients who are mildly obese. Furthermore, the sample size of the obese group in our study was smaller than the number of people who were normal weight or overweight, which may also contribute to the difference in results between the obese population and the rest of the subjects.

Numerous metabolic processes can be regulated by THs. Low THs within the normal range can lead to higher prevalence of metabolic disturbance such as T2DM and metabolic syndrome [24, 30, 31]. When assessing the relationship between THs and IR, as reflected by HOMA-IR, FT4 was found to be negatively associated with IR in euthyroid subjects [6, 25, 32]. In contrast, no significant link was found between FT4 and HOMA-IR in a random sample in Palanga [7]. The paradoxical conclusion may be due to the different characteristics of study population among these studies. For example, the study in Palanga did not exclude those with abnormal thyroid function or blood glucose level. New indexes that reflect sensitivity to THs, such as TFQI, TSHI, and TT4RI, have been raised. Reduced insulin sensitivity, as measured by the Matsuda index, was linked to poor central and peripheral TH sensitivity in prepubertal obese children, according to Domenico Corica's research [33]. Our findings are quite similar to those of Cheng et al., who found a strong positive correlation between central thyroid sensitivity index and TyG based on data from the National Health and Nutrition Examination Survey (NHANES) [34]. Specifically, the association between TFQI and TyG did not show any significance whether or not confounding factors were adjusted in their study. Notably, our study proved a both positive and significant association between TFQI and TyG in patients whose BMI are within 24–28 (*p* < 0.01), but not in those who are overweight. Yang et al. involved euthyroid patients with or without diabetes and found the existence of subclinical hypothyroidism as an indicator of elevated HOMA-IR in the normoglycemic group. However, no linear association was found between TFQI and IR, as reflected by HOMA-IR, in the entire study population [35].

Insulin is the base on which HOMA-IR is calculated, yet it is not commonly evaluated in clinical settings. There have been many studies using newly proposed indexes that are both accurate and clinically convenient to represent IR. A study involved 82 Brazilian subjects explored whether TyG was superior to HOMA-IR in reflecting IR. From Spearman analysis, the correlation between the level of IR obtained from the hyperglycemic clamp test showed a greater association with TyG (*r* = −0.64; *p* < 0.001) than with HOMA-IR (*r* = −0.51; *p* < 0.001) [36]. The narrow sample size and the study population's geographic restrictions, however, might partially undermine the above conclusion's universality. Zhang et al. pointed out that when evaluating the accuracy of TyG in quantifying IR, it showed 96% sensitivity of HIEC as a reference and 99% specificity of HOMA-IR as a reference [28]. AIP was considered to have a good performance when representing IR [18]. Specifically, Yin et al. found a stronger link between AIP and HOMA-IR in men than in women [37]. Whether that will influence the accuracy of conclusion in our study remains unknown. From a cross-sectional study involving 1370 hypothyroid patients, an upward trend was discovered between TSH and AIP [38]. Unlike our study which was based on euthyroid subjects, this study broadened the population to those with abnormal thyroid function and illustrated the relationship between TSH and IR in them. In the state of THs-resistant, high THs and TSH coexist. It is uncertain, though, if higher TSH in these people indicates diminished TH sensitivity. Unlike TyG and AIP which are only based on glucolipid metabolism parameters, the calculation of METS-IR additionally incorporated the effect of BMI, which is a crucial contributor of IR. In a cross-sectional investigation with 260 patients without diabetes, the effectiveness of METS-IR as an IR marker was shown. Meanwhile, significantly higher METS-IR was found in patients with overt hypothyroidism than in euthyroid patients [39]. All of the three indexes are clinically handy, as the parameters required for calculation are easily accessible. Notably, they are all TG-based, which is a volatile parameter and can be easily affected by diet. Calculating them using only the results of TG from a single blood test might affect the reliability of their prediction of IR.

Thyroid hormone receptors (THRs) are highly expressed in liver, through which TH may regulate hepatic lipid metabolism and eventually influencing IR. TH promotes liver uptake and β-oxidation of free fatty acid as well as hepatic lipogenesis. Impaired sensitivity to thyroid hormones was found to be linked with elevated blood triglyceride level, leading to elevated circulating levels of FFA, which is the breakdown product of triglycerides [40]. Excessive free fatty acid inhibits insulin signaling through the synthesis of ceramide [41]. Ceramides have been demonstrated to block insulin signaling in mice by activating protein phosphatase 2A, which in turn causes AKT, an insulin-stimulated kinase essential for signal transduction, to become inactive [42]. When FFA exceeds the storage capacity of intrinsic adipose tissue, fat accumulates in non–adipose tissues such as liver, skeletal muscle, pancreas, and myocardium, forming ectopic lipid deposits and IR [43]. By altering the expression activity of related transcription factors including SREBP1C, LXRs, and ChREBP, TH also stimulates hepatic lipogenesis. In patients with steatotic liver disease, accumulation of lipid intermediate, such as diacylglycerol in liver, could induce hepatic IR. This may be due to the insulin signaling changes in the liver [44]. Consistently, THRβ-specific agonist was found to reduce hepatic steatosis and improve hepatic IR in rats [45].

THs are involved in the control of mitochondrial activity as well. In rats' skeletal muscle, adipose tissue, and liver, T3-mediated mitochondrial autophagy may increase mitochondrial protein synthesis, O_2_ consumption, and maximal respiratory capacity. Mitochondrial dysfunction in insulin target organs was probably a contributor to IR and the progression of T2DM [46]. When ROS production in skeletal muscle is elevated due to mitochondrial failure, c-Jun N-terminal kinase is activated, phosphorylating the insulin receptor substrate and inhibiting insulin signaling [47]. In the skeletal muscle of people with IR status, there was a decline in the efficiency of mitochondrial oxidative phosphorylation, mitochondrial enzyme activity, mitochondrial content, and fatty acid oxidation [48–50]. Chennamsetty et al. found that insulin sensitivity was caused by mitochondrial malfunction and a lower basal metabolic rate in the adipose tissue of NAT1 gene-deficient mice [51]. It was also shown that increased mitochondrial oxidative phosphorylation in adipose tissue following gastric bypass surgery could restore the decreased insulin sensitivity in diabetic individuals [52].

The HPT axis typically regulates the amount of THs in the bloodstream through a negative feedback regulatory system [53]. High THs and high TSH coexist in those with central TH resistance due to a compromised pituitary feedback set point [54]. Leptin and the thyroid axis have a complex and dual connection, as shown by in vitro experiments [55]. Elevated leptin levels stimulated by increased TSH may lead to hyperinsulinemia and IR [56]. TSH also induces intracellular oxidative stress and cyclophilin D acetylation, which contribute to an imbalance between endothelin-1 and endothelial nitric oxide synthase and, ultimately, failure of endothelium-dependent vasodilation [57]. This may lead to the defect of skeletal muscle perfusion and impaired insulin-mediated glucose uptake.

There are some limitations in our current study. Firstly, the causality between TH sensitivity and the levels of IR could not be verified by the cross-sectional study. Secondly, only participants from China were included, and this might limit the universality of our study. Thirdly, TyG, AIP, and METS-IR are non-insulin-based IR indexes; therefore, their accuracy in predicting IR may be lower than insulin-based direct measures of IR such as HOMA-IR and the quantitative insulin sensitivity check index (QUICKI). Also, lack of information on diet and lifestyle may lower their IR-predicting ability.

In conclusion, we found that reduced sensitivity to TH was associated with high levels of IR in euthyroid adults of normal weight and overweight. In obese patients, no significant correlation was found between sensitivity to THs and IR. Since IR is the basis of many chronic metabolic diseases, our study provides insight for further exploring the pathogenesis of metabolic syndrome as well as guidance for its treatment.

## Figures and Tables

**Table 1:** Baseline characteristics of the study population.

	Overall *N* = 32,478	Normal *N* = 20,200	Overweight *N* = 10,178	Obesity *N* = 2100
Age, years	43.2 ± 13.3	41.4 ± 13.0	46.4 ± 13.2	44.7 ± 13.0
SBP, mmHg	121 ± 17	116 ± 16	127 ± 16	134 ± 16
DBP, mmHg	71.6 ± 11.3	68.6 ± 10.3	75.5 ± 11.1	80.5 ± 11.5
Hypertension, *n* (%)	27,609 (85.0%)	18,370 (90.9%)	7882 (77.4%)	1357 (64.6%)
FBG, mmol/L	4.87 ± 1.17	4.69 ± 0.95	5.13 ± 1.39	5.33 ± 1.55
HbA1c, %	5.58 ± 0.71	5.46 ± 0.59	5.73 ± 0.82	5.91 ± 0.93
UA, μmol/L	340 ± 90	314 ± 80	378 ± 89	409 ± 95
TG, mmol/L	1.42 ± 1.19	1.15 ± 0.82	1.80 ± 1.47	2.13 ± 1.82
TC, mmol/L	4.90 ± 0.91	4.86 ± 0.89	4.98 ± 0.94	4.99 ± 0.95
HDL-C, mmol/L	1.39 ± 0.37	1.51 ± 0.38	1.22 ± 0.28	1.14 ± 0.25
LDL-C, mmol/L	3.00 ± 0.85	2.90 ± 0.82	3.16 ± 0.86	3.22 ± 0.89
TyG	8.42 ± 0.62	8.22 ± 0.53	8.71 ± 0.61	8.91 ± 0.63
AIP	−0.120 ± 0.716	−0.368 ± 0.627	0.250 ± 0.661	0.477 ± 0.646
METS-IR	35.0 ± 8.6	30.3 ± 5.8	41.0 ± 5.1	50.9 ± 6.3
FT3, pg/mL	3.27 ± 0.349	3.22 ± 0.345	3.34 ± 0.341	3.40 ± 0.340
FT4, ng/dL	1.25 ± 0.154	1.25 ± 0.152	1.26 ± 0.156	1.26 ± 0.159
TSH, μIU/mL	2.01 ± 0.89	2.00 ± 0.89	2.01 ± 0.90	2.04 ± 0.89
TFQI	0.011 ± 0.383	0.011 ± 0.385	0.011 ± 0.379	0.011 ± 0.383
TSHI	1.91 ± 0.46	1.90 ± 0.46	1.91 ± 0.46	1.94 ± 0.45
TT4RI	19.4 ± 8.7	19.3 ± 8.7	19.4 ± 8.6	19.9 ± 8.8

*Note:* Data were expressed as mean ± SD or number (proportion).

Abbreviations: AIP, Atherogenic Index of Plasma; BMI, body mass index; DBP, diastolic blood pressure; FPG, fasting plasma glucose; FT3, free triiodothyronine; FT4, free thyroxine; HbA1c, glycated hemoglobin; HDL-C, high-density lipoprotein cholesterol; LDL-C, low-density lipoprotein cholesterol; METS-IR, Metabolic Score for Insulin Resistance; SBP, systolic blood pressure; TC, total cholesterol; TFQI, Thyroid Feedback Quantile-based Index; TG, triglycerides; TSH, thyroid-stimulating hormone; TSHI, TSH index; TT4RI, Thyrotroph T4 Resistance Index; TyG, triglyceride-glucose index; UA, uric acid.

**Table 2:** Study population's TyG, AIP, and METS-IR by quartiles of TFQI, TSHI, and TT4RI levels.

	TFQI					TSHI						TT4RI				
	Q1	Q2	Q3	Q4	*p* value	Q1		Q2	Q3	Q4	*p* value	Q1	Q2	Q3	Q4	*p* value
*BMI* *<* *25*																
AIP	−0.440 ± 0.627	−0.380 ± 0.624	−0.346 ± 0.626	−0.308 ± 0.625	*p* < 0.001	−0.423 ± 0.626		−0.382 ± 0.630	−0.362 ± 0.615	−0.307 ± 0.632	*p* < 0.001	−0.416 ± 0.628	−0.382 ± 0.633	−0.364 ± 0.616)	−0.312 ± 0.628	*p* < 0.001
TyG	8.17 ± 0.53	8.21 ± 0.52	8.24 ± 0.53	8.27 ± 0.53	*p* < 0.001	8.18 ± 0.54		8.21 ± 0.53	8.23 ± 0.52	8.28 ± 0.53	*p* < 0.001	8.18 ± 0.54	8.21 ± 0.53	8.23 ± 0.53	8.27 ± 0.53	*p* < 0.001
METS-IR	30.1 ± 4.7	30.3 ± 4.7	30.5 ± 4.8	30.3 ± 8.2	*p* < 0.001	30.2 ± 4.7		30.3 ± 4.8	30.4 ± 4.8	30.3 ± 8.2	*p*=0.084	30.2 ± 4.7	30.3 ± 4.8	30.4 ± 4.8	30.3 ± 8.2	*p*=0.370

*25 ≤ BMI < 29.9*																
AIP	0.213 ± 0.650	0.222 ± 0.659	0.257 ± 0.657	0.309 ± 0.674	*p* < 0.001	0.217 ± 0.657		0.251 ± 0.654	0.246 ± 0.666	0.287 ± 0.665	*p*=0.002	0.220 ± 0.660	0.259 ± 0.651	0.243 ± 0.675	0.280 ± 0.659	*p*=0.011
TyG	8.68 ± 0.58	8.69 ± 0.60	8.72 ± 0.61	8.77 ± 0.63	*p* < 0.001	8.69 ± 0.60		8.71 ± 0.60	8.71 ± 0.62	8.75 ± 0.61	*p* < 0.001	8.69 ± 0.60	8.71 ± 0.60	8.71 ± 0.62	8.75 ± 0.61	*p*=0.006
METS-IR	40.9 ± 4.5	40.9 ± 4.7	41.0 ± 4.7	41.4 ± 6.4	*p* < 0.001	40.9 ± 4.6		41.1 ± 4.6	41.0 ± 4.7	41.2 ± 6.4	*p*=0.268	40.9 ± 4.7	41.1 ± 4.6	41.0 ± 4.7	41.2 ± 6.4	*p*=0.296

*BMI ≥ 30*																
AIP	0.405 ± 0.678	0.437 ± 0.618	0.521 ± 0.636	0.544 ± 0.640	*p* < 0.001		0.409 ± 0.659	0.512 ± 0.666	0.453 ± 0.624	0.533 ± 0.628	*p*=0.007	0.419 ± 0.667	0.513 ± 0.655	0.443 ± 0.626	0.532 ± 0.628	*p*=0.010
TyG	8.85 ± 0.62	8.86 ± 0.60	8.93 ± 0.61	9.00 ± 0.67	*p* < 0.001		8.84 ± 0.62	8.94 ± 0.64	8.87 ± 0.61	9.00 ± 0.63	*p* < 0.001	8.85 ± 0.62	8.93 ± 0.63	8.87 ± 0.63	8.99 ± 0.62	*p* < 0.001
METS-IR	50.4 ± 6.0	50.4 ± 6.0	51.0 ± 6.0	51.6 ± 7.0	*p*=0.004		50.4 ± 6.0	50.9 ± 6.2	50.7 ± 6.0	51.5 ± 6.8	*p*=0.056	50.5 ± 6.0	50.8 ± 6.1	50.8 ± 6.1	51.4 ± 6.8	*p*=0.084

*Note:* Data were expressed as mean ± SD. *p* values for TyG and AIP were calculated using ANOVA test, and *p* values for METS-IR were calculated using Kruskal–Wallis test.

Abbreviations: AIP, Atherogenic Index of Plasma; BMI, body mass index; METS-IR, Metabolic Score for Insulin Resistance; Q1, quartile 1; Q2, quartile 2; Q3, quartile 3; Q4, quartile 4; TFQI, Thyroid Feedback Quantile-based Index; TSHI, TSH index; TT4RI, Thyrotroph T4 Resistance Index; TyG, Triglyceride-Glucose Index.

**Table 3:** Linear regression analysis for the association between IR levels and thyroid hormone sensitivity indices.

Variables	TyG		AIP		METS-IR		
	Standardized *β* (*t*)	*p* value	Standardized *β* (*t*)	*p* value	Standardized *β* (*t*)	*p* value	
*BMI < 25*							
TFQI	0.096 (11.788)	< 0.001	0.093 (9.370)	< 0.001	0.103 (1.357)	0.175	
TSHI	0.089 (13.178)	< 0.001	0.099 (12.003)	< 0.001	0.187 (2.985)	0.003	
TT4RI	0.089 (13.009)	< 0.001	0.101 (12.144)	< 0.001	0.203 (3.176)	0.001	

*25* *≤* *BMI* *<* *29.9*						
TFQI	0.071 (5.157)	< 0.001	0.053 (3.307)	< 0.001	0.174 (1.633)	0.103	
TSHI	0.067 (5.884)	< 0.001	0.068 (5.143)	< 0.001	0.301 (3.442)	< 0.001	
TT4RI	0.066 (5.741)	< 0.001	0.070 (5.205)	< 0.001	0.319 (3.590)	< 0.001	

*BMI* *≥* *30*							
TFQI	0.097 (3.119)	0.002	0.081 (2.354)	0.019	0.249 (1.048)	0.295	
TSHI	0.084 (3.170)	0.002	0.073 (2.515)	0.012	0.231 (1.141)	0.254	
TT4RI	0.083 (3.071)	0.002	0.074 (2.481)	0.013	0.238 (1.156)	0.248	

*Note:* Linear regression analysis for the association between IR levels (dependent variable) and thyroid hormone sensitivity indices (independent variables) in euthyroid population. Age, sex, BMI, HbA1c, UA, and hypertension were adjusted.

Abbreviations: AIP, Atherogenic Index of Plasma; BMI, body mass index; METS-IR, Metabolic Score for Insulin Resistance; TFQI, Thyroid Feedback Quantile-based Index; TSHI, TSH index; TT4RI, Thyrotroph T4 Resistance Index; TyG, Triglyceride-Glucose Index.

## Data Availability

The data that support the conclusions of this study are not publicly available due to privacy or ethical restrictions. Requests to access the datasets should be directed to Guang Wang, wangguangcy@ccmu.edu.cn.

## Nomenclature

BMI: Body mass index
DBP: Diastolic blood pressure
FBG: Fasting blood glucose
FT3: Free triiodothyronine
FT4: Free thyroxine
HbA1c: Glycated hemoglobin
HDL-C: High-density lipoprotein cholesterol
HOMA-IR: Homeostasis model assessment of insulin resistance
IR: Insulin resistance
LDL-C: Low-density lipoprotein cholesterol
TC: Total cholesterol
TG: Triglyceride
TH: Thyroid hormone
TSH: Thyroid-stimulating hormone
TFQI: Thyroid Feedback Quantile-based Index
TSHI: Thyrotropin index
TT4RI: Thyrotropin Thyroxine Resistance Index
UA: Uric acid

## References

[1] DeFronzo R. A., Ferrannini E. (1991). Insulin Resistance: A Multifaceted Syndrome Responsible for NIDDM, Obesity, Hypertension, Dyslipidemia, and Atherosclerotic Cardiovascular Disease. *Diabetes Care*.

[2] Eckel R. H., Grundy S. M., Zimmet P. Z. (2005). The Metabolic Syndrome. *The Lancet*.

[3] Sun H., Chang X., Bian N. (2022). Adipose Tissue Insulin Resistance Is Positively Associated With Serum Uric Acid Levels and Hyperuricemia in Northern Chinese Adults. *Frontiers in Endocrinology*.

[4] Diamanti-Kandarakis E., Dunaif A. (2012). Insulin Resistance and the Polycystic Ovary Syndrome Revisited: An Update on Mechanisms and Implications. *Endocrine Reviews*.

[5] Chen W., Cai W., Hoover B., Kahn C. R. (2022). Insulin Action in the Brain: Cell Types, Circuits, and Diseases. *Trends in Neurosciences*.

[6] Amouzegar A., Kazemian E., Gharibzadeh S., Mehran L., Tohidi M., Azizi F. (2015). Association Between Thyroid Hormones, Thyroid Antibodies and Insulin Resistance in Euthyroid Individuals: A Population-Based Cohort. *Diabetes and Metabolism*.

[7] Kazukauskiene N., Podlipskyte A., Varoneckas G., Mickuviene N. (2021). Insulin Resistance in Association With Thyroid Function, Psychoemotional State, and Cardiovascular Risk Factors. *International Journal of Environmental Research and Public Health*.

[8] Jostel A., Ryder W. D., Shalet S. M. (2009). The Use of Thyroid Function Tests in the Diagnosis of Hypopituitarism: Definition and Evaluation of the TSH Index. *Clinical Endocrinology*.

[9] Yagi H., Pohlenz J., Hayashi Y., Sakurai A., Refetoff S. (1997). Resistance to Thyroid Hormone Caused by Two Mutant Thyroid Hormone Receptors Beta, R243Q and R243W, With Marked Impairment of Function that Cannot Be Explained by Altered in Vitro 3,5,3′-Triiodothyroinine Binding Affinity. *Journal of Clinical Endocrinology and Metabolism*.

[10] Laclaustra M., Moreno-Franco B., Lou-Bonafonte J. M. (2019). Impaired Sensitivity to Thyroid Hormones is Associated With Diabetes and Metabolic Syndrome. *Diabetes Care*.

[11] Wan H., Yu G., Xu S. (2023). Central Sensitivity to Free Triiodothyronine With MAFLD and Its Progression to Liver Fibrosis in Euthyroid Adults. *Journal of Clinical Endocrinology and Metabolism*.

[12] Wei Y., Li X., Cui R., Liu J., Wang G. (2024). Associations Between Sensitivity to Thyroid Hormones and Insulin Resistance in Euthyroid Adults With Obesity. *Frontiers in Endocrinology*.

[13] Bonora E., Targher G., Alberiche M. (2000). Homeostasis Model Assessment Closely Mirrors the Glucose Clamp Technique in the Assessment of Insulin Sensitivity: Studies in Subjects With Various Degrees of Glucose Tolerance and Insulin Sensitivity. *Diabetes Care*.

[14] Guerrero-Romero F., Simental-Mendía L. E., González-Ortiz M. (2010). The Product of Triglycerides and Glucose, a Simple Measure of Insulin Sensitivity: Comparison With the Euglycemic-Hyperinsulinemic Clamp. *The Journal of Cinical Endocrinology and Metabolism*.

[15] Dobiásová M., Frohlich J. (2001). The Plasma Parameter Log (TG/HDL-C) as an Atherogenic Index: Correlation With Lipoprotein Particle Size and Esterification Rate in Apob-Lipoprotein-Depleted Plasma (FER (HDL)). *Clinical Biochemistry*.

[16] Bello-Chavolla O. Y., Almeda-Valdes P., Gomez-Velasco D. (2018). METS-IR, a Novel Score to Evaluate Insulin Sensitivity, Is Predictive of Visceral Adiposity and Incident Type 2 Diabetes. *European Journal of Endocrinology*.

[17] Tao L. C., Xu J. N., Wang T. T., Hua F., Li J. J. (2022). Triglyceride-Glucose Index as a Marker in Cardiovascular Diseases: Landscape and Limitations. *Cardiovascular Diabetology*.

[18] Tan M. H., Johns D., Glazer N. B. (2004). Pioglitazone Reduces Atherogenic Index of Plasma in Patients With Type 2 Diabetes. *Clinical Chemistry*.

[19] Yao Z., Ding X., Gao X. (2021). Irisin as a Potential Biomarker Associated With Myocardial Injuries in Patients With Severe Hypothyroidism. *International Journal of Endocrinology*.

[20] Liu J., Zhang L., Fu J., Wang Q., Wang G. (2021). Circulating Prolactin Level Is Increased in Metabolically Healthy Obesity. *Endocrine Connections*.

[21] Mohajan D., Mohajan H. K. (2023). Body Mass Index (BMI) Is a Popular Anthropometric Tool to Measure Obesity Among Adults. *Journal of Innovations in Medical Research*.

[22] Zhou B., Carrillo-Larco R. M., Danaei G. (2021). Worldwide Trends in Hypertension Prevalence and Progress in Treatment and Control From 1990 to 2019: A Pooled Analysis of 1201 Population-Representative Studies With 104 Million Participants. *The Lancet*.

[23] Tsou M. T., Yun C. H., Lin J. L. (2020). Visceral Adiposity, Pro-Inflammatory Signaling and Vasculopathy in Metabolically Unhealthy Non-Obesity Phenotype. *Diagnostics*.

[24] Mehran L., Amouzegar A., Bakhtiyari M. (2017). Variations in Serum Free Thyroxine Concentration Within the Reference Range Predicts the Incidence of Metabolic Syndrome in Non-Obese Adults: A Cohort Study. *Thyroid*.

[25] Ghergherehchi R., Hazhir N. (2015). Thyroid Hormonal Status Among Children With Obesity. *Therapeutic Advances in Endocrinology and Metabolism*.

[26] Taylor P. N., Richmond R., Davies N. (2016). Paradoxical Relationship Between Body Mass Index and Thyroid Hormone Levels: A Study Using Mendelian Randomization. *Journal of Clinical Endocrinology and Metabolism*.

[27] Ahmed B., Sultana R., Greene M. W. (2021). Adipose Tissue and Insulin Resistance in Obese. *Biomedicine & Pharmacotherapy*.

[28] Zhang R., Dong S. Y., Wang F. (2018). Associations Between Body Composition Indices and Metabolic Disorders in Chinese Adults: A Cross-Sectional Observational Study. *Chinese Medical Journal*.

[29] Agrawal S., Wang M., Klarqvist M. D. R. (2022). Inherited Basis of Visceral, Abdominal Subcutaneous and Gluteofemoral Fat Depots. *Nature Communications*.

[30] Mehran L., Delbari N., Amouzegar A., Hasheminia M., Tohidi M., Azizi F. (2022). Reduced Sensitivity to Thyroid Hormone is Associated With Diabetes and Hypertension. *Journal of Clinical Endocrinology and Metabolism*.

[31] Kim B. J., Kim T. Y., Koh J. M. (2009). Relationship Between Serum Free T4 (FT4) Levels and Metabolic Syndrome (MS) and Its Components in Healthy Euthyroid Subjects. *Clinical Endocrinology*.

[32] van Tienhoven-Wind L. J., Dullaart R. P. (2015). Low-Normal Thyroid Function and Novel Cardiometabolic Biomarkers. *Nutrients*.

[33] Corica D., Licenziati M. R., Calcaterra V. (2022). Central and Peripheral Sensitivity to Thyroid Hormones and Glucose Metabolism in Prepubertal Children With Obesity: Pilot Multicenter Evaluation. *Endocrine*.

[34] Cheng H., Hu Y., Zhao H. (2023). Exploring the Association Between Triglyceride-Glucose Index and Thyroid Function. *European Journal of Medical Research*.

[35] Yang W., Jin C., Wang H., Lai Y., Li J., Shan Z. (2023). Subclinical Hypothyroidism Increases Insulin Resistance in Normoglycemic People. *Frontiers in Endocrinology*.

[36] Garduño-Garcia J. d J., Alvirde-Garcia U., López-Carrasco G. (2010). TSH and Free Thyroxine Concentrations Are Associated With Differing Metabolic Markers in Euthyroid Subjects. *European Journal of Endocrinology*.

[37] Yin B., Wu Z., Xia Y., Xiao S., Chen L., Li Y. (2023). Non-Linear Association of Atherogenic Index of Plasma With Insulin Resistance and Type 2 Diabetes: A Cross-Sectional Study. *Cardiovascular Diabetology*.

[38] Demirhan S., Polat O., Mert M. (2023). The Relationship Between Tsh Levels, Thyroid Autoantibodies and Atherogenic Index of Plasma, Ast to Platelet Ratio Index Score, and Fibrosis 4 Index in Patients With Hypothyroidism. *Acta Endocrinologica*.

[39] Tunc Karaman S. (2023). Insulin Resistance in Non-Diabetic Hypothyroid Patients: A Critical Examination of METS-IR as a Diagnostic Marker. *Current Medical Research and Opinion*.

[40] Liu Y., Ma M., Li L. (2022). Association Between Sensitivity to Thyroid Hormones and Dyslipidemia in Patients With Coronary Heart Disease. *Endocrine*.

[41] Lee S. H., Park S. Y., Choi C. S. (2022). Insulin Resistance: From Mechanisms to Therapeutic Strategies. *Diabetes & Metabolism Journal*.

[42] Chaurasia B., Tippetts T. S., Mayoral Monibas R. (2019). Targeting a Ceramide Double Bond Improves Insulin Resistance and Hepatic Steatosis. *Science*.

[43] Rosqvist F., Iggman D., Kullberg J. (2014). Overfeeding Polyunsaturated and Saturated Fat Causes Distinct Effects on Liver and Visceral Fat Accumulation in Humans. *Diabetes*.

[44] Scoditti E., Sabatini S., Carli F., Gastaldelli A. (2024). Hepatic Glucose Metabolism in the Steatotic Liver. *Nature Reviews Gastroenterology & Hepatology*.

[45] Sinha R. A., Singh B. K., Yen P. M. (2018). Direct Effects of Thyroid Hormones on Hepatic Lipid Metabolism. *Nature Reviews Endocrinology*.

[46] Emily Tubbs SpC, Robert M., Bendridi N. (2018). Disruption of Mitochondria-Associated Endoplasmic Reticulum Membranes (Mams) Integrity Contributes to Muscle Insulin Resistance in Mice and Humans. *Diabetes*.

[47] Lowell B. B., Shulman G. I. (2005). Mitochondrial Dysfunction and Type 2 Diabetes. *Science*.

[48] Kelley D. E., He J., Menshikova E. V., Ritov V. B. (2002). Dysfunction of Mitochondria in Human Skeletal Muscle in Type 2 Diabetes. *Diabetes*.

[49] Pinti M. V., Fink G. K., Hathaway Q. A., Durr A. J., Kunovac A., Hollander J. M. (2019). Mitochondrial Dysfunction in Type 2 Diabetes Mellitus-An Organ-Based Analysis. *American Journal of Physiology, Endocrinology and Metabolism*.

[50] Befroy D. E., Petersen K. F., Dufour S. (2007). Impaired Mitochondrial Substrate Oxidation in Muscle of Insulin-Resistant Offspring of Type 2 Diabetic Patients. *Diabetes*.

[51] Chennamsetty I., Coronado M., Contrepois K. (2016). Nat1 Deficiency Is Associated With Mitochondrial Dysfunction and Exercise Intolerance in Mice. *Cell Reports*.

[52] Hansen M., Lund M. T., Gregers E. (2015). Adipose Tissue Mitochondrial Respiration and Lipolysis Before and After a Weight Loss by Diet and RYGB. *Obesity*.

[53] Ortiga‐Carvalho T. M., Chiamolera M. I., Pazos-Moura C. C., Wondisford F. E. (2016). Hypothalamus-Pituitary-Thyroid Axis. *Comprehensive Physiology*.

[54] Cao B., Li K., Ke J., Zhao D. (2024). Impaired Sensitivity to Thyroid Hormones Is Associated With the Change of Abdominal Fat in Euthyroid Type 2 Diabetes Patients: A Retrospective Cohort Study. *Journal of Diabetes Research*.

[55] Menendez C., Baldelli R., Camina J. (2003). TSH Stimulates Leptin Secretion by a Direct Effect on Adipocytes. *Journal of Endocrinology*.

[56] Friedman J. M. (2019). Leptin and the Endocrine Control of Energy Balance. *Nature Metabolism*.

[57] Liu X., Du H., Chai Q. (2018). Blocking Mitochondrial Cyclophilin D Ameliorates TSH-Impaired Defensive Barrier of Artery. *Redox Biology*.

[58] Cui R., Wei Y., Liu J., Wang Y., Wang G. (2023). Impaired Sensitivity to Thyroid Hormones Is Associated With High Insulin Resistance Levels in the Euthyroid Chinese Population. *Research Square*.

